# Atorvastatin Accelerates Alveolar Bone Loss in Type 1 Diabetic Rats Submitted to Periodontitis

**DOI:** 10.1590/0103-6440202406100

**Published:** 2024-10-28

**Authors:** Gisele Barreto Angelino, Karysia Veras, Delane Gondim Viana, Karuza Maria Alves Pereira, Renata Leitão, Gerly Anne de Castro Brito, Hellíada Vasconcelos Chaves, Mirna Marques, Paula Goes

**Affiliations:** 1 Post-Graduate Program in Morphological Science, Federal University of Ceara, Fortaleza, Ceara, Brazil; 2 Post-Graduate Program in Health Science, Federal University of Ceara, Fortaleza, Ceara, Brazil; 3 Department of Morphology, Federal University of Ceara, Fortaleza, Ceara, Brazil; 4 School of Medicine, Federal University of Ceara, Fortaleza, Ceara, Brazil; 5 Department of Pathology and Legal Medicine, Federal University of Ceara, Fortaleza, Ceara, Brazil; 6 Department of Pathology and Legal Medicine, Federal University of Ceara, Fortaleza, Ceara, Brazil

**Keywords:** Periodontitis, Diabetes, Atorvastatin, Alveolar Bone Loss

## Abstract

Periodontal bone loss is potentiated by diabetes. Despite the beneficial anti-inflammatory and antiresorptive effects of Atorvastatin (ATV) on periodontitis, it has been reported to increase the risk of diabetes, which may modify the course of periodontal disease. Therefore, this study aimed to evaluate the effect of ATV on alveolar bone in rats with periodontitis and diabetes. For this, 72 Wistar rats were divided into groups: Naïve (N) not submitted to any procedure; Experimental periodontitis (EP) group submitted to ligature-induced periodontitis; diabetes mellitus (DM), submitted to EP and receiving single dose of streptozotocin (60 mg/kg, i.p.) after 12 hours of fasting; and ATV DM, submitted to EP and DM and receiving orally 27 mg/kg of ATV, 30 minutes before ligature placement, and continued daily until the 11^th^ day. Animals from EP and DM received saline solution 0.9% as placebo. Glycemic levels measured in all animals and then were euthanized. Maxillae were collected for macroscopic, micro-tomographic, and microscopic analyses. DM caused intense bone loss (60%), characterized by a reduction in trabecular thickness and bone volume. DM reduced osteoblasts, increasing osteoclast counts, and induced an inflammatory infiltrate in the periodontium. ATV was found ineffective in protecting bone in diabetic rats, exacerbating bone loss by 21%. Additionally, ATV significantly increased blood glucose levels. In summary, ATV did not prevent alveolar bone loss or modulate inflammation in DM animals undergoing EP. ATV also increased blood glucose levels in these animals. Therefore, the systemic use of ATV in uncontrolled diabetic conditions should be carefully evaluated.

## Resumo

A perda óssea causada pela periodontite pode ser potencializada pela diabetes. Apesar dos efeitos benéficos, anti-inflamatórios e antirreabsortivos, da Atorvastatina (ATV) na periodontite tem sido relatado o aumento para o risco de diabetes o que pode modificar o curso da doença periodontal. Portanto, este estudo objetiva avaliar o efeito da ATV no osso alveolar de ratos com periodontite e diabetes. Pra tanto, 72 ratos Wistar foram divididos em 4 grupos: Naïve (N), periodontite experimental (EP), diabetes mellitus (DM) e ATV. A DM foi induzida através da estreptozotocina (60 mg/kg-ip. em dose única) após 12 horas de jejum. Em seguida todos os animais foram submetidos a EP. A ATV foi administrada (27 mg/kg, v.o) 30 minutos antes da ligadura e diariamente por 11 dias. Animais do grupo N não foram submetidos a nenhuma intervenção. Após 1 dias de EP, todos os animais foram submetidos a medição da glicemia e em seguida eutanasiados. As maxilas foram coletadas para análises macroscópicas, micro-tomográficas e microscópicas. A DM causou intensa perda óssea (60%) marcada pela redução da espessura trabecular e do volume ósseo (p<0.05). Foi observado uma redução significante em osteoblastos com aumento da contagem de osteoclastos, bem como aumento do infiltrado inflamatório no periodonto. A ATV não foi capaz de proteger o osso alveolar nos animais diabéticos, aumentando a perda óssea em 21%. A ATV causou aumento significante dos níveis de glicose no sangue (20%). Em suma, a ATV não protegeu a perda do osso alveolar e não modulou a inflamação da EP nos animais diabéticos. A ATV aumentou os níveis de glicose no sangue desse grupo de animais. Portanto, o uso sistêmico de ATV em diabéticos não-controlados deve ser cuidadosamente avaliada.

## Introduction

Periodontitis is the second most prevalent disease in the oral cavity, characterized by chronic inflammation resulting from the host's immune response to microorganisms present in the dental biofilm. This chronic inflammation leads to the deterioration of dental support tissues ^1^. Additionally, the onset and progression of this disease are often influenced by various environmental and genetic factors, with diabetes mellitus being a notable factor among them ^2^.

Diabetes Mellitus (DM) is an endocrine disorder characterized by the body's inability to utilize glucose adequately due to insufficient levels of the hormone insulin, or due to difficulty in using the produced insulin, resulting in elevated blood glucose levels. DM is mainly divided into: Type 2 Diabetes Mellitus (T2DM), one of the most common metabolic disorders, and it is caused by defective insulin secretion and/or inability of insulin-sensitive tissues to respond; or T1DM, an autoimmune disorder, where insulin-producing β cells are destroyed. Despite the lower prevalence, T1DM is the most severe form of DM, requeiring multiple daily insulin injections and even with excellent glucose control, patients are at significant risk for developing complications ^3^.

DM is associated with a range of potential long-term complications affecting blood vessels, nerves, kidneys, and bone. Diabetes and chronic periodontitis have long been recognized as biologically interconnected conditions. DM stands as one of the primary risk factors for periodontitis, exacerbating inflammation and promoting bone loss ^4^. Conversely, periodontitis can induce insulin resistance, further impairing glycemic control ^5^. The relationship between Type 1 diabetes and Periodontitis is less clear and further studies are needed ^3^.

Hence, in cases of heightened inflammation, particularly as induced by DM, the utilization of pharmacological adjuvants may present a promising approach to enhance periodontal treatment. In this context, Atorvastatin (ATV) emerges as a noteworthy candidate due to its pleiotropic effects ^6^. Our research group has demonstrated that systemic ATV exhibits anti-inflammatory and anti-resorptive properties in experimental periodontitis in rats ^7^ ^,^ ^8^, as well as in animals subjected to glucocorticoid-induced osteoporosis ^9^.

Despite the beneficial effects of statins, randomized controlled trials have indicated that when administered systemically, Atorvastatin (ATV) can lead to an increase in blood sugar levels ^10^. Consequently, we hypothesized that ATV-induced hyperglycemia might exacerbate inflammation and bone loss in diabetic animals with periodontitis. Therefore, the objective of this study was to investigate the impact of Atorvastatin on the alveolar bone tissue of diabetic rats subjected to periodontitis.

## Materials and Method

### Experimental Design

This was a randomized, controlled, and blinded study. The experimental protocols were conducted following the guidelines outlined in ARRIVE (Animal Research: Reporting In Vivo Experiments) for the ethical use of experimental animals. Experiments started only after obtaining approval from the Institutional Animal Use Ethics Committee, under protocol number 150/17.

### Animal Selection

A total of 72 male Wistar rats (*Rattus norvegicus*) were used in this study, all obtained from our own facility. These rats weighted approximately 200 grams and were 12 weeks old. The animals were housed in appropriate cages, with six animals per cage, and were provided with a well-balanced commercial diet and unrestricted access to water. They were kept under consistent environmental conditions, including a 12-hour light/dark cycle and a room temperature of 22°C throughout the experiment.

The sample size calculation was based on previous studies conducted by our research group ^7^ ^,^ ^8^ ^,^ ^11^ ^,^ ^12^ in which significant differences were observed, with alveolar bone loss greater than 2mm serving as the primary outcome variable. The number of animals required was 6 per group in order to provide a power calculation of 80%, and significant level of p < 0.05. Furthermore, the animals were randomly assigned into the experimental groups, using a block randomization strategy.

The experimental groups consisted of 6 animals per group and were as follows:

1. Naive (N) group: Animals in this group did not undergo any experimental procedures.
2. Experimental periodontitis (EP) group: Animals in this group were induced with ligature-induced periodontitis and received 2ml/kg of saline solution 0,9% orally. Five days before ligature placement they received a single dose of citrate buffer solution.
3. Type 1 Diabetes mellitus (DM) group: Animals in this group were made diabetic by administering a single dose of Streptozotocin (60 mg/kg, intraperitoneally). Five days later, they were subjected to periodontitis induction and received 2ml/kg of saline solution 0,9% orally.
4. Atorvastatin (ATV) group: Animals in this group, were submitted to both diabetes and periodontitis, and received 27 mg/kg of Atorvastatin via oral gavage ^7^ 30 minutes before ligature placement and continued daily until the 11^th^ day.

### Type 1 Diabetes Mellitus (DM) Model

Diabetes was induced by administering a single intraperitoneal injection of Streptozotocin (STZ) (CAYMAN® Life Science Research, Solon, OH, USA) at a dose of 60 mg/kg, diluted in citrate buffer (0.01 M, pH 4.5). This injection was carried out following a 12-hour fasting period (FURMAN, 2015). Animals in EP groups received a single intraperitoneal injection of citrate buffer solution. Twenty minutes later, all animals were provided with unrestricted access to food and water.

After 5 days, clinical manifestations associated with diabetes, such as polyuria, polydipsia, polyphagia, and weight loss, were observed and further confirmed through a fasting glucose assay. Blood samples were collected on the 5^th^ experimental day, the day of EP induction, to measure plasma glucose levels. Animals with glycemic levels exceeding 200 mg/dl ^13^ five days following the STZ injection were considered diabetic and included in the study. Additionally, blood glucose levels were assessed on the day of euthanasia (16^th^ experimental day) to evaluate the glycemic status following the treatment.

### Ligature-induced periodontitis model

To induce inflammatory bone loss, we employed the ligature-induced periodontitis model, a method based on our previous studies ^7^ ^,^ ^8^ ^,^ ^11^ ^,^ ^12^. This model entails the placement of a 3.0 nylon suture thread around the upper left 2nd molar of a rat, performed under anesthesia (80 mg/kg of Ketamine + 10 mg/kg of Xylazine, intraperitoneally). A surgical knot was positioned on the vestibular side of the tooth. The animals were monitored for 11 days until euthanasia. Animals in the N group did not undergo ligature model. Animals in the EP and DM groups received 2 ml/kg of 0.9% sterile saline solution via gavage 30 minutes before ligature placement and daily until the 11^th^ day.

### Treatment with Atorvastatin (ATV)

Atorvastatin (Vast®, Pfizer, São Paulo, BR) was administered orally at a dosage of 27 mg/kg for a duration of 11 days in the ATV group ^7^ ^,^ ^8^ ^,^ ^11^. The treatment with ATV was initiated 30 minutes prior to the induction of periodontitis and continued daily until euthanasia. Animals in the groups not receiving ATV were administered 2 ml/kg of 0.9% saline solution via oral gavage.

### Morphometric analysis of alveolar bone loss

At the conclusion of the experiment, the animals had their maxillae removed and fixed in 10% formalin for a duration of 48 hours. Subsequently, the maxillae were carefully dissected and divided into two halves. To distinguish bone tissue from teeth, the dissected specimens were stained with 1% methylene blue, a method consistent with our previous studies ^7^ ^,^ ^8^ ^,^ ^11^.

To assess bone resorption, both halves were photographed, and the resulting images were analyzed using the IMAGE J® Software. Alveolar bone loss (ABL) was quantified by measuring the area from the cusp tip to the remaining bone edge and then subtracting this value from the corresponding area in the contralateral hemimaxillae. This area was initially calculated in pixels and subsequently converted to mm^2^, using a millimeter pattern affixed to the side of the jaws, following the method outlined in GOES et al. ^8^.

### Micro-tomographic analysis of bone tissue

The same specimens utilized for macroscopic analysis were subjected to cone beam microtomography (µCT) using the Skyscan 1172 system from Bruker, Kontich, Belgium. The X-ray generator operated at an accelerated potential of 50 kV, with a beam current of 200 µA and an exposure time of 560 ms per projection. This configuration produced images with a voxel size of 6x6x6 µm. Three-dimensional models were generated using dedicated software (Data Viewer®, version 1.5.0, Bruker, Kontich, Belgium).

In the interproximal region, we measured the distance between the alveolar bone crest and the cementum-enamel junction (ABL-CEJ). In the furcation area, Trabecular Thickness (Tb.Th) and Bone Volume (BV/TV) were evaluated. All measurements were carried out using specialized software (CT-Analyser®, version 1.13.5.1+, Bruker, Kontich, Belgium), following the methodology as outlined in SOUSA et al. ^9^.

### Histopathological analyzes of bone tissue

In another series of experiments, following euthanasia, the maxillae were harvested for histopathological analysis. The specimens were initially fixed in 10% buffered formalin for a period of 48 hours. Subsequently, they underwent demineralization with 10% buffered EDTA over approximately 30 days. The halves of the maxillae were embedded in paraffin, sectioned into 4 µm thick slices, and then stained with Hematoxylin and Eosin (H&E).

For microscopic analysis, we focused on the region between the 1st and 2nd molars and evaluated inflammatory aspects, including the presence and intensity of cellular infiltrates and the state of preservation of the alveolar process and cement. We assigned scores ranging from 0 to 3 based on the intensity of these findings ^14^. To quantify the number of osteoblasts per bone perimeter (N.Oc/B.Pm), we selected four fields within the same interproximal region, observed under 400x magnification. Image J Software (NIH, Bethesda, USA) was employed for this analysis ^12^. Histologically, osteoblasts were characterized as cuboidal or slightly elongated cells forming a continuous cell layer over the bone surface, each with a single nucleus

### Tartrate Resistant Acid Phosphatase (TRAP) Staining

Additional 4 µm sections were obtained from the paraffin block and used for Tartrate Resistant Acid Phosphatase (TRAP) staining. We selected four fields in the interproximal region between the 1st and 2nd molars, observed under 400x magnification, to quantify the number of osteoclasts per bone perimeter (N.Oc/B.Pm) using Image J software (NIH, Bethesda, USA) ^12^. Osteoclasts were identified based on positive tartrate-resistant acid phosphatase staining and their characteristic appearance as multinucleated giant cells adjacent to the bone surface

### Glycemic control

We assessed the capillary blood glucose levels of the animals using a glucometer. The measurements were taken at three key points: first, at the outset of the study (day 0) before administering STZ; second, on the 5th day (following DM induction) to confirm the presence of diabetes in the animals (as defined by blood glucose levels exceeding 200 mg/dl) ^13^; and third, on the day of euthanasia (day 16), to assess the animals' glycemic control at the conclusion of the study.

### Statistical analysis

The data are reported as either mean ± standard error of the mean or as median (range), as appropriate. To compare the means, we employed ANOVA followed by the Tukey test, while the Kruskal-Wallis and Dunn tests were utilized to compare medians. A p-value of < 0.05 was considered indicative of statistically significant differences. All calculations and analyses were conducted using GraphPad Prism 8.0 software (GraphPad, Inc., San Diego, CA, USA). It's worth noting that all protocols and analyses were conducted in a blinded manner.

## Results

### ATV accelerated periodontal bone loss in type 1 diabetic animals

**Figure 1:**
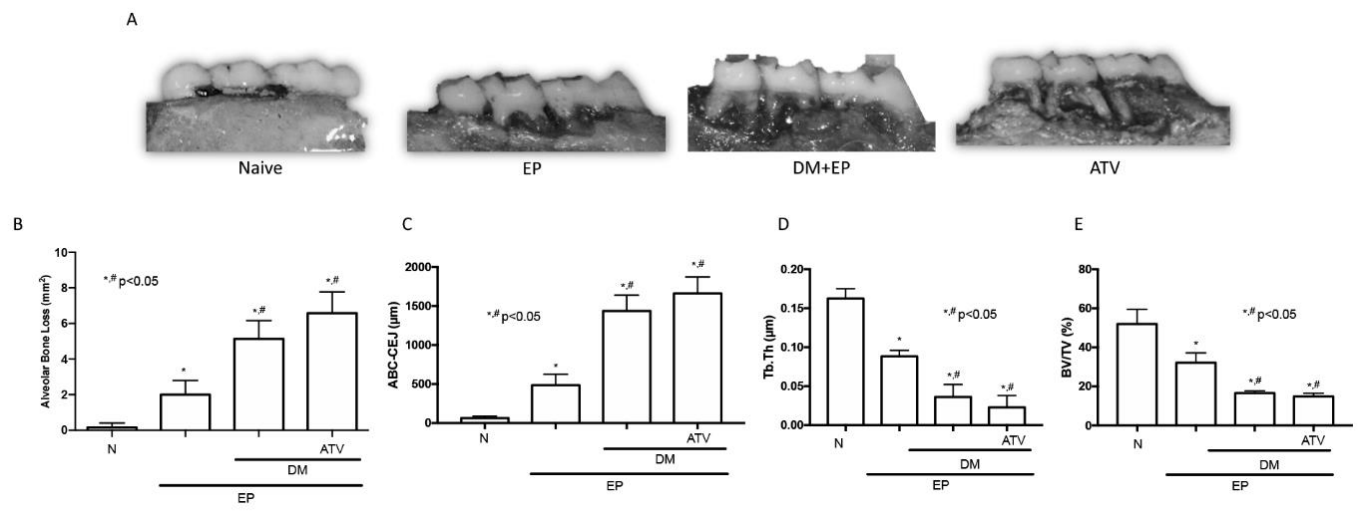
ATV accelerated periodontal bone loss in diabetic animals. (A) Macroscopic aspect of rat maxilla. (B) Analysis of alveolar bone loss. Microtomographic analyses of (C) the distance between alveolar bone crest and cementum-enamel junction, (D) Trabecular thickness, (E) Bone volume/ Total volume. Bars represent the mean±standard error of mean of 6 rats per group. *Significant difference compared to the Naive group. #Significant difference compared to the EP group. ANOVA followed by Tukey test. P<0.05.

Periodontitis caused significant bone loss (ure 1B), furcation lesions and root exposures when compared to hemimaxillae of N group (Figure 1A) (p<0.05), confirming the effectiveness of the model. In the other hand, DM potentiated bone loss by 60% (Figure 1A, 1B) (p<0.05). ATV was not able to protect the bone tissue. An increase of 21% on bone loss was seen after ATV treatment (Figure 1A, 1B).

Micro-CT analyses confirmed the macroscopic findings. EP increased distance between the alveolar bone crest to cementum-enamel junction (ABC-CEJ) in the interproximal region (Figure 1C) and reduced the trabecular thickness (Figure 1D) and bone volume (Figure 1E) (p<0.05). DM caused a greater increase on ABC-CEJ by 66% (Figure 1C), reduced the trabecular thickness (-62%) (Figure 1D) as well as bone volume (-51,5%) (Figure 1E). ATV, in turn, did not reverse any of bone micro-CT features seen in DM group (Figure 1C, 1D, 1E). Taking together, in diabetic conditions, ATV failed to reduce bone resorption.

### ATV neither modulated inflammation nor improved bone cell count in type 1 diabetic rats with periodontitis

**Figure 2:**
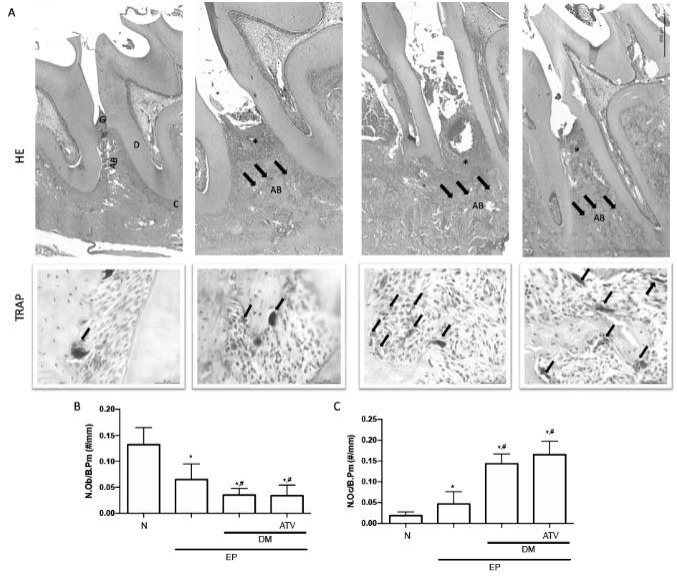
ATV did not modulate inflammation and affected bone cell count in diabetic animals with periodontitis**.** Histomorphometric analysis of hemimaxilla of rats in the interproximal site with HE and TRAP staining. (B) Osteoblast count/mm of bone and (C) Osteoclast count/mm of bone. In HE staining was used 40x magnification. G= Gingiva; D= Dentin; C= Cement; AB= Alveolar Bone. Inflammatory infiltrate is indicated with asterisks and bone resorption with arrows. In TRAP staining arrow indicates osteoclasts in 400x magnification. Bars represent the mean±standard error of mean of 6 rats per group. *Significant difference compared to the Naive group. #Significant difference compared to the EP group. ANOVA followed by Tukey test. P<0.05.

Table 1 shows the histological analysis of the periodontium of animals. The interproximal region of the rats submitted to periodontitis showed intense inflammatory infiltrate, partial destruction of cementum and alveolar process when compared to the Naive group (Figure 2A) (p<0.05). Diabetic animals submitted to periodontitis revealed an intense infiltrate of inflammatory cells and marked alveolar resorption as well as severe cementum destruction (Figure 2A) (p<0.05). The group treated with ATV had an inflammatory infiltrate like the one seen in DM group (Figure 2A). These findings show that ATV was not effective in reducing inflammation during diabetes.

**Table 1:** Effect of Atorvastatin on Histopathological Alterations in the periodontium of diabetic rats submitted to periodontitis.

Histological Analysis (scores)	Naïve	EP	DM+EP	ATV
	0 (0 -0)	2 (1 -3) *	3 (2-3) *	3 (2 - 3) *

Values ​​established as median (extreme values), considering 6 rats per group. EP = Experimental Periodontitis; DM = Diabetes Mellitus; ATV = Atorvastatin. * Significant difference compared to the Naive group. Kruskal-Wallis followed by Dunn’s test.

Considering the bone cell count, as expected periodontitis significantly increased the osteoclasts number (Figure 2A, 2C) with reduction on osteoblast count (Figure 2B). DM increased even more the number of osteoclasts (71%) (Figure 2A, 2C) together with a reduction by half on osteoblast count (Figure 2B) (p<0.05). ATV did not change the amount of these bone cells as seen in DM group (Figure 2A-C). In this way, we can speculate that ATV cannot promote bone formation under a high glucose level environment.

### ATV increases glycemia in animals with type 1 diabetes and periodontitis

**Figure 3:**
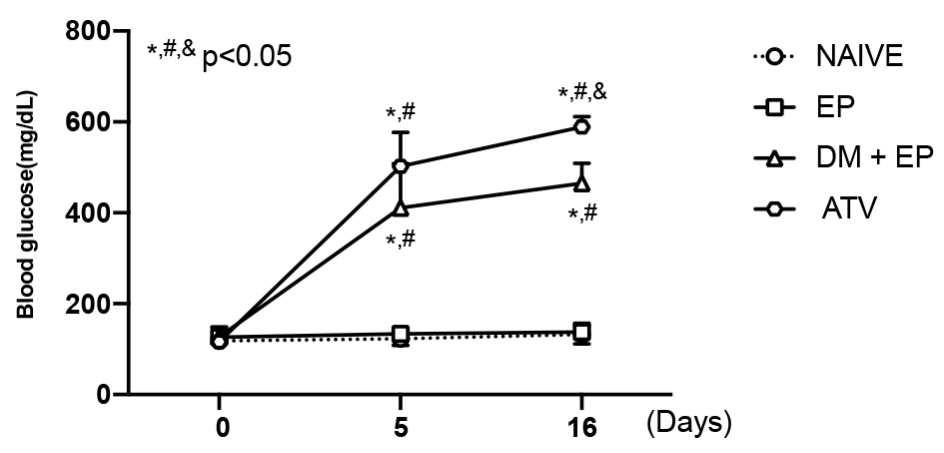
ATV increases glycemia in animals with diabetes and periodontitis**.** Analysis of capillary blood glucose at the beginning, fifth day and end of the study. Points represent the mean±standard error of mean of 6 rats per group. *Significant difference compared to the Naive group. #Significant difference compared to the EP group. &Significant difference compared to the DM+EP group. (ANOVA followed by Tukey test). P<0.05.

All animals initiated the experiment with normal glycemia. The ones submitted to DM showed a significant increase of blood glycemic levels, 5 days after the disease induction. By the end of the experiment, it was seen that the animals with periodontitis maintained its normal blood glycemic rates similar to the animals from N group. The rats with diabetes showed a greater increase of blood glucose confirming the diabetic status. However, the animals treated with ATV presented an even higher glycemia (+20%) when compared to DM group (p<0.05) (Figure 3). Indicating that ATV does not contribute to glycemic control and therefore, it may increase the risk for diabetes.

## Discussion

In this study, we observed that ligature-induced periodontitis successfully replicated the key features of human periodontitis ^15^. Notably, diabetes mellitus (DM) exacerbated alveolar bone loss, as evidenced through macroscopic examination and micro-CT analysis. Micro-CT results showed a reduction in trabecular thickness and bone volume, an increase in osteoclast counts, and a decrease in the number of osteoblasts in the alveolar bone. Additionally, diabetic rats exhibited a heightened inflammatory infiltrate in the periodontium. These findings collectively affirm the detrimental impact of hyperglycemia on bone tissue, aligning with prior research ^16^. However, it's worth noting that treatment with ATV failed to reverse the bone loss and inflammation induced by diabetes. Intriguingly, ATV also led to a 20% increase in blood glucose levels in diabetic animals, suggesting a potential association with an elevated risk of diabetes when using this medication.

ATV is known for its pleiotropic effects, including anti-inflammatory and bone anabolic properties. Both its local and systemic usage have been demonstrated to be beneficial in the context of periodontitis ^7^ ^,^ ^8^. In a previous study from our group, we have evaluated the effect of ATV for 11 days administered by gavage. At 27 mg/kg, ATV inhibited alveolar bone loss (ABL) by 56%. Histopathological analysis showed that ATV 27 mg/kg prevented ABL, cementum resorption, and inflammatory cell infiltration, induced by ligature. ATV (27 mg/kg) prevented reduction on bone-specific alkaline phosphatase (BALP) serum levels, as well as leukocytosis and did not affect either kidney or liver function nor body mass weight ^8^. Meanwhile, in rats with glucocorticoid-induced osteoporosis submitted to periodontitis, ATV at 27 mg/kg decreased bone loss, reduced myeloperoxidase (MPO), TNF-α, IL-1β, IL-6, and IL-8, and increased IL-10, glutathione (GSH), superoxide dismutase (SOD), and catalase (CAT) levels. ATV at 27 mg/kg also reduced RANKL and DKK-1 and increased OPG, WNT10b, and β-catenin expressions and BALP activity. Taken together these finding have already shown the beneficial effect of ATV use during periodontitis ^9^.

Notably, in diabetic patients, local delivery of ATV has shown effectiveness in reducing bone resorption ^17^ ^,^ ^18^ ^,^ ^19^. However, the systemic administration of ATV in diabetic individuals has been associated with a higher risk of developing diabetes ^10^. This study aimed to investigate the systemic administration of ATV in diabetic rats subjected to periodontitis, and to the best of our knowledge, this marks the first instance of such an investigation.

The results of this study revealed that ATV increased blood glucose levels in diabetic animals. Several mechanisms have been proposed to explain the association between statins and the development of diabetes. It has been reported that ATV reduces the translocation of glucose transporter 4 (GLUT4) into the cell membrane. GLUT4 is a facilitative transporter crucial for peripheral insulin-mediated glucose uptake, and the reduction in its translocation can lead to insulin resistance ^20^. Additionally, ATV may contribute to an increased risk of diabetes by inhibiting the phosphorylation events or small G-protein activation required to initiate insulin signal transduction ^21^. Furthermore, ATV has been shown to inhibit adipocyte differentiation, and undifferentiated adipocytes do not secrete insulin-sensitizing hormones. An excess of these undifferentiated cells can increase insulin resistance ^20^. Moreover, it's known that increased intracellular calcium concentration is necessary to trigger insulin secretion through the opening of calcium channels. Altered function or levels of calcium channels may thus influence glucose homeostasis. Studies have demonstrated that ATV may impair calcium channels ^20^. Additionally, other mechanisms, such as the role of microRNAs in insulin levels and sensitivity, have been investigated, and ATV has been shown to induce the hepatic expression of microRNA-33a, which could lead to decreased insulin secretion ^22^. Collectively, these mechanisms help explain why ATV can contribute to hyperglycemia, as observed in this study, consequently increasing the risk of diabetes.

This study also demonstrated that ATV was unable to reverse or ameliorate the bone loss induced by DM and experimental periodontitis. Elevated glucose levels, as observed in the ATV group, exert toxic effects on various cells and tissues, including bone. Poor glycemic control has been associated with an increased relative risk of hip fracture ^23^, which is accompanied by a reduction in bone mineral density ^24^ along with low bone turnover ^25^. In alveolar bone, a prior study aligns with our findings, as it demonstrated that simvastatin was incapable of protecting bone in animals with DM subjected to EP ^26^. The potential mechanism by which hyperglycemia induces bone loss involves alterations in both bone formation and resorption ^2^.

Our results indicated that ATV was not able to reverse the reduction on the number of osteoblasts seen in diabetic animals with periodontitis. This decrease in osteoblasts can be attributed to the impact of hyperglycemia on the expression of transcription factors regulating osteoblast differentiation, such as Runx2 ^27^. High glucose levels have been shown to reduce the migratory potential and chemotaxis of osteoblasts, which leads to inefficient migration to areas requiring repair, resulting in irregular mineralization and bone fragility ^28^. Furthermore, hyperglycemia can diminish alkaline phosphatase activity, impair mineralized matrix formation, and promote osteoblast apoptosis ^29^.

This study uncovered that ATV treatment in diabetic rats subjected to periodontitis led to an increase in the number of osteoclasts. Elevated glucose levels have been shown to directly promote osteoclast differentiation and activation through the upregulation of macrophage-colony stimulating factor, receptor activator of nuclear factor kappa-B ligand (RANKL), and vascular endothelial growth factor ^30^ ^,^ ^31^. Additionally, hyperglycemic conditions have been associated with heightened expression of cathepsin K, a marker of osteoclast activity ^30^.

Furthermore, the treatment with Atorvastatin (ATV) failed to modulate the inflammation induced by ligature-induced periodontitis in diabetic rats. Rats with diabetes mellitus are known to exhibit a higher degree of inflammation and a more persistent inflammatory response following periodontitis ^32^. This heightened inflammation in diabetic rats can be attributed to various factors. Elevated glucose levels stimulate the increased production of chemokines that induce neutrophil recruitment, as well as the generation of reactive oxygen species (ROS). Macrophages, under hyperglycemic conditions, intensify the production of interleukin-1 and tumor necrosis factor (TNF). Additionally, dendritic cells are influenced by diabetes, promoting the generation of Th1 or Th17 lymphocytes while reducing the formation of regulatory T cells. It is well-established that the exacerbation of the inflammatory response has a detrimental effect on bone metabolism, ultimately contributing to bone loss.

Despite the intriguing findings, this study is not without its limitations. This study does not provide an in-depth exploration of the molecular and biological mechanisms underlying bone resorption in diabetic animals treated with ATV. The use of contralateral hemimaxillae may be considered, once periodontitis can cause systemic effects in the animals, however its use also corroborates with the principal of 3Rs, in order to minimize the number of animals used in the experiment. It is worth considering that the effects of ATV should also be assessed in other models of diabetes mellitus to further validate our data. Moreover, in humans, Type 1 DM would not use only statins but rather a combination with exogenous insulin, therefore further studies must be performed to evaluate the effect of Atorvastatin in animals with DM under insulin therapy.

In summary, these findings suggest that ATV may contribute to the dysregulation of glycemic levels induced by diabetes. The adverse effects of ATV in diabetic animals subjected to periodontitis are characterized by increased bone resorption and inflammation, along with a reduction in bone formation. It's important to note that further studies are required to corroborate these findings.
